# The Effects of the COVID-19 Pandemic on the Work and Financial Outcomes of Older Black and Hispanic Adults

**DOI:** 10.1093/geroni/igab046.085

**Published:** 2021-12-17

**Authors:** Kendra Jason, Dawn Carr, Zhao Chen

**Affiliations:** 1 UNC Charlotte, Charlotte, North Carolina, United States; 2 Florida State University, Tallahassee, Florida, United States; 3 The University of Arizona, Tucson, Arizona, United States

## Abstract

This study investigates how older Black and Hispanic adults’ work engagement is impacted by the effects of COVID-19. Using intersectionality and cumulative (dis)advantage as complementing theoretical frameworks, data from the Health and Retirement Study, and series of logistic regression models, we measure work engagement changes pre- and post- COVID-19. Preliminary findings suggest that net of other controls, there were no substantial or significant reductions in resilience as the result of COVID-19 itself for any racial/ethnic group. White older adults, and to some degree Hispanics older adults, experienced erosion in resilience related to financial hardships, but the resilience of Black older adults remained stable in the face of increased hardship. Future work in this area will improve our limited understanding of older Black and Hispanic adults’ experiences of managing and coping with COVID-19- related work and financial risks– information that will be critical for planning intervention and support services.

